# Error in Byline

**DOI:** 10.1001/jamanetworkopen.2026.42881

**Published:** 2026-10-09

In the Original Investigation titled “Inhaled Corticosteroids Continuation in the First Trimester and Pregnancy Outcomes in Women With Asthma,”^1^ published August 24, 2026, author Hoon Kim’s academic degrees should have appeared as MD, PhD instead of MS, PhD. This article has been corrected.^1^

## References

[1] Suh B, Cho Y, Choi EY, . Inhaled corticosteroids continuation in the first trimester and pregnancy outcomes in women with asthma. JAMA Netw Open. 2026;9(8):e2630781. doi:10.1001/jamanetworkopen.2026.30781 42635984 PMC13504433

